# Joint effect of physical capacity and physical activity predicts depression progression in middle-aged and older Chinese adults

**DOI:** 10.7189/jogh.16.04093

**Published:** 2026-03-27

**Authors:** Xiong Ding, Hui Zhou, Ruolin Zhang, Weiqiang Wu, Yanjuan Chen

**Affiliations:** 1 Center for Endemic Disease Control, Chinese Center for Disease Control and Prevention, Harbin Medical University, Harbin, China; 2 Xiangya School of Nursing, Central South University, Changsha, China; 3 Harvard T.H. Chan School of Public Health, Harvard University, Boston, USA; 4 Department of Cardiology, Second Affiliated Hospital of Shantou University Medical College, Shantou, China; 5 Department of Endocrinology, Second Affiliated Hospital of Shantou University Medical College, Shantou, China

## Abstract

**Background:**

Physical capacity (PC) and physical activity (PA) are key behavioural and physiological determinants of mental health, yet their joint effect on the progression of depression remains unclear. Understanding how these domains interact within a biobehavioural framework may help identify modifiable factors to prevent depression in ageing populations. Using a quadrant-based biobehavioural approach, we aimed to examine the joint effect of PC and PA on the progression of depressive symptoms.

**Methods:**

We drew data for a cohort of participants surveyed in the China Health and Retirement Longitudinal Study from 2011 to 2018. PC was assessed by the Short Physical Performance Battery, and PA by weekly metabolic equivalent of task minutes. Participants were categorised into four PC-PA quadrants: ‘can do, do do’, ‘can do, don’t do’, ‘can’t do, do do’, and ‘can’t do, don’t do’. Depressive symptoms were measured repeatedly with the 10-item Center for Epidemiologic Studies Depression Scale. We used linear mixed-effects models to estimate trajectories of depression across quadrants.

**Results:**

Compared with the ‘can’t do, don’t do’ group, participants in the ‘can do, do do’ (*P* = 0.001) and ‘can do, don’t do’ (*P* < 0.001) quadrants had significantly lower depressive symptom levels at baseline. Longitudinal trajectories of depressive symptoms differed by sex: changes over time were largely similar across PC-PA quadrants among men, while women with preserved PC showed a steeper increase in depressive symptoms over time despite lower baseline levels.

**Conclusions:**

Preserved PC is associated with a more favourable mental health status in middle-aged and older adults, including those with low PA. Sex-specific differences in longitudinal depressive symptom patterns highlight the need to consider PC when monitoring mental health in ageing populations.

Depression is a substantial global public health challenge, contributing to increased risks of comorbid conditions, healthcare costs, and mortality rates [1,2]. The World Health Organization (WHO) estimates that depression affects over 300 million people globally, with a particularly heavy burden among ageing populations [3]. In China, the prevalence of depression among middle-aged and older adults continues to rise, with rates reported to be as high as 23.61% [4]. Identifying modifiable behavioural and physiological factors that influence the onset and progression of depression is, therefore, crucial for developing effective prevention and intervention strategies.

Physical activity (PA) and physical capacity (PC) are recognised behavioural determinants of mental health. The former typically encompasses routine daily activities, while the latter refers to the capability (*e.g. *strength, balance, and endurance) to perform such activities [5]. A meta-analysis of 49 prospective cohort studies involving 266 939 participants demonstrated a 17% reduction in depression risk with higher PA levels, irrespective of age or region [6]. Similarly, a systematic review of over one million individuals found that lower cardiorespiratory fitness, a marker of physical inactivity, was associated with a 75% increased risk of developing depression [7]. Numerous studies have also shown that higher levels of PC are associated with lower depressive symptoms [8].

PC and PA may influence depression through overlapping, yet distinct pathways. The former reflects underlying physiological reserve and functional independence, which are closely linked to self-efficacy and perceived control, while the latter represents actual behavioural engagement that may affect neurobiological processes related to mood regulation. Importantly, adequate PC may be a prerequisite for sustaining meaningful activity over time, while low capacity may limit the psychological benefits of activity even when participation is present. This interdependence suggests that examining PC and PA jointly, rather than in isolation [9].

Despite increasing evidence linking PC and PA individually to depression, few studies have examined their joint effects within a unified framework that captures both behavioural engagement and physiological capacity. Koolen *et al*. [10] proposed a biobehavioural approach that uses PC-PA quadrants in clinical populations with chronic obstructive pulmonary disease. This framework classified individuals with the condition based on combinations of PC and PA levels into four distinct statuses: ‘can do, do do’, ‘can do, don’t do’, ‘can’t do, do do’, and ‘can’t do, don’t do’, to better capture the heterogeneity in their physical functioning [10]. Subsequent studies have applied this approach to outcomes such as fall risk and mortality, suggesting its potential utility for broader health risk assessment beyond clinical settings [11,12]. However, its relevance for mental health outcomes in population-based samples remains largely unexplored.

Using data from a nationally representative longitudinal cohort, we wanted to extend this biobehavioural quadrant framework to middle-aged and older adults in China and examines its association with longitudinal trajectories of depressive symptoms. By integrating behavioural and physiological measures within said framework, we sought to identify high-risk groups and inform targeted behavioural medicine interventions for depression prevention.

## METHODS

### Study design and participants

We retrieved data from the China Health and Retirement Longitudinal Study (CHARLS) [13], a nationally representative longitudinal survey initiated in 2011. The CHARLS employed a multistage stratified probability proportional sampling method to recruit 17 708 individuals aged 45 years and older from 450 communities and 150 counties across 28 provinces in China. Through face-to-face interviews, the CHARLS staff collected data on sociodemographic characteristics, lifestyle factors, and self-reported health status and recorded them in CHARLS database. Follow-up assessments were conducted every 2–3 years. The CHARLS study was approved by the Institutional Review Board at Peking University (IRB No. 00001052-11015), and all participants provided written informed consent. We report our findings per the STROBE guidelines and confirm its alignment with the Journal of Global Health’s GRABDROP guidelines. (Checklists S1 and S2 in the **Online Supplementary Document**).

We first conducted a cross-sectional analysis. In the CHARLS, PA data were collected from a random subsample of approximately half of the baseline participants. Accordingly, we excluded participants <45 years of age whose data were collected because household members (*e.g.* spouses) were also surveyed and age information was re-verified during follow up, as well as those with missing data on baseline PC, PA, or depression. For the longitudinal analysis, we further excluded participants with incomplete depression scores during 2013, 2015, and 2018 follow-ups.

### Assessment of depression

Depressive symptoms in the CHARLS were measured using the 10-item Center for Epidemiologic Studies Depression Scale (CESD-10), which has been validated and has demonstrated strong reliability in older Chinese populations [14]. The CESD-10 consists of 10 items, each with four response options: ‘rarely or none of the time’ (<1 day/week), ‘some days’ (1–2 days/week), ‘occasionally’ (3–4 days/week), and ‘most of the time’ (5–7 days/week). Responses ae scored from 0 to 3, with higher scores indicating more frequent depressive symptoms, and with two positively worded items reverse scored to align with the overall scoring system. The total CESD-10 score thus ranges from 0 to 30, with higher scores reflecting more severe depressive symptoms [15]. For the cross-sectional analysis, we assessed depressive symptoms using the CESD-10 score from the baseline (2011) survey. For the longitudinal analysis, we evaluated depression progression using repeated measurements of the CESD-10 score across follow-up waves (2011, 2013, 2015, and 2018).

### Assessment of physical activity

In the CHARLS, PA was assessed using a modified International Physical Activity Questionnaire (IPAQ) [16]. Participants reported the intensity of their weekly PA (categorised as vigorous, moderate, and walking) and its duration (<10, 10–29, 30–119, 120–239, and ≥240 minutes/day), and frequency (number of days, 0–7). We assigned the following median value of each interval to quantify activity duration: 0 minutes (<10 minutes/day), 20 minutes (10–29 minutes/day), 75 minutes (30–119 minutes/day), 180-minute (120–239 minutes/day), and 240 minutes (≥240 minutes/day). We then calculated the total PA for each intensity level in metabolic equivalent of task (MET) minutes/week, calculated as follows: walking (3.3 × minutes × days), moderate intensity (4.0 × minutes × days), and vigorous intensity (8.0 × minutes × days). The sum of MET-minutes for all intensities provided a composite measure of each participant’s total weekly PA [17].

### Assessment of physical capacity

PC was measured using the Short Physical Performance Battery (SPPB), which includes gait speed, a progressive balance test (semi-tandem, tandem, and side-by-side stands), and the time to complete five chair stands to assess physical function [18]. Trained CHARLS interviewers demonstrated each test before the participants’ attempt, and recorded gait speed twice, and the better of the two performances was used for scoring. Each item is scored on a scale from 0 to 4, with higher scores indicating better physical performance; the overall SPPB score ranges from 0 (worst) to 12 (best) and is calculated by summing the individual component scores [19]. We assessed PC and PA at baseline and treated them as baseline exposures to examine how initial functional and behavioural status relates to subsequent trajectories of depressive symptoms.

### Covariates

We selected the following covariates based on previous research [20,21]:

– demographics: age (years), sex (man or woman), residence (urban or rural), marital status (with or without a spouse);

– socioeconomic factors: education level (primary school and below, secondary school, or college and above), medical insurance (no or yes), and endowment insurance (no or yes);

– lifestyle factors: smoking history (no or yes), alcohol consumption in the last year (no or yes), sleep duration (1–5, 6–8, and >8 hours), and body mass index category (<18.5, 18.5–24.0, 24.0–28.0, and ≥28.0 kg/m^2^);

– comorbidities: number of comorbid conditions (none, 1, or ≥2 conditions), cognitive function (score), and peak expiratory flow (L/min).

Cognitive function was assessed using a composite cognition score derived from four domains (orientation, memory, computation, and drawing ability) which were then summed to yield a total cognition score ranging from 0 to 31, with higher scores indicating better cognitive performance [22].

### Statistical analysis

We examined continuous variables for normality using the Shapiro-Wilk test and visually inspected histograms with normal curves. Given the large sample size and the approximately symmetric, unimodal distributions we observed, we presented continuous variables means and standard deviations (SDs), and categorical variables as percentages and frequencies.

We determined optimal threshold values for predicting depression risk based on PC (SPPB score) and PA (MET minutes/week) using receiver operating characteristic curves with the Youden index, calculating sensitivity and specificity to assess the predictive accuracy of these thresholds. Based on the derived thresholds, we classified participants into four PC-PA quadrants: preserved PC and preserved PA (‘can do, do do’), preserved PC and low PA (‘can do, don’t do’), low PC and low PA (‘can’t do, don’t do’), and low PC and preserved PA (‘can’t do, do do’). We examined differences in baseline characteristics across the four quadrants using the χ^2^ test or one-way ANOVA, as appropriate. Given that both depression and PA are influenced by sex [23,24], we conducted stratified analyses for men and women.

For the cross-sectional analysis, we used generalised linear models to estimate regression coefficients (*β*) and 95% confidence intervals (95% CI) for each PC-PA quadrant, with the ‘can’t do, don’t do’ quadrant serving as the reference. We explored longitudinal relationships between PC-PA quadrants and depression progression through linear mixed-effects models. These models included repeated measures of depression scores (including baseline scores) as outcome variables, with PC-PA quadrants, time (years since baseline), their interaction (PC-PA quadrants × time), and covariates as fixed effects. The regression coefficients for PC-PA quadrants represented differences in baseline depression scores compared to the reference group; time coefficients indicated the overall rate of change in depression scores during follow-up; and interaction coefficients (PC-PA quadrants × time) reflected differences in the rate of change in depression scores between quadrants. We adjusted covariates for age, sex, marital status, education, residence, medical insurance, endowment insurance, smoking history, drinking status, sleep duration, comorbidity, body mass index, cognition scores, and peak expiratory flow. Additionally, we included random slopes for time to account for individual differences in the rate of depression changes during follow up and conducted stratified analyses by age and residence.

We performed two sensitivity tests to strengthen the robustness of the associations. First, we redefined PA thresholds according to the latest WHO guidelines [25] and set a new PC threshold with an SPPB score of 9 [26]. Second, we excluded participants with baseline cardiovascular or pulmonary diseases to minimise potential confounding, as these conditions may affect both PA and depression.

We performed all statistical analyses using Stata, version 17.0 software (StataCorp LLC., College Station, Texas, USA). All *P* values were two-sided, and a *P*-value <0.05 was considered as statistically significant.

## RESULTS

For the cross-sectional analysis, we excluded 12 097 participants aged <45 years (n = 648), missing data on PC (n = 3608), PA (n = 7812), or depression (n = 29), leaving a sample of 5611 participants. For the longitudinal analysis, we removed an additional 2022 participants for a total of 3589 (Figure S1 in the **Online Supplementary Document**).

The 5611 participants at baseline had a mean age of 59.4 years (SD = 9.5) and 45.9% were men. The optimal thresholds for predicting depression were determined as 8 points for PC across both sexes, while the thresholds for PA were 8032.5 MET minutes/week for men and 9600 MET min/week for women (Table S1 in the **Online Supplementary Document**).

### Baseline distribution of PC and PA quadrants

**Figure 1:**
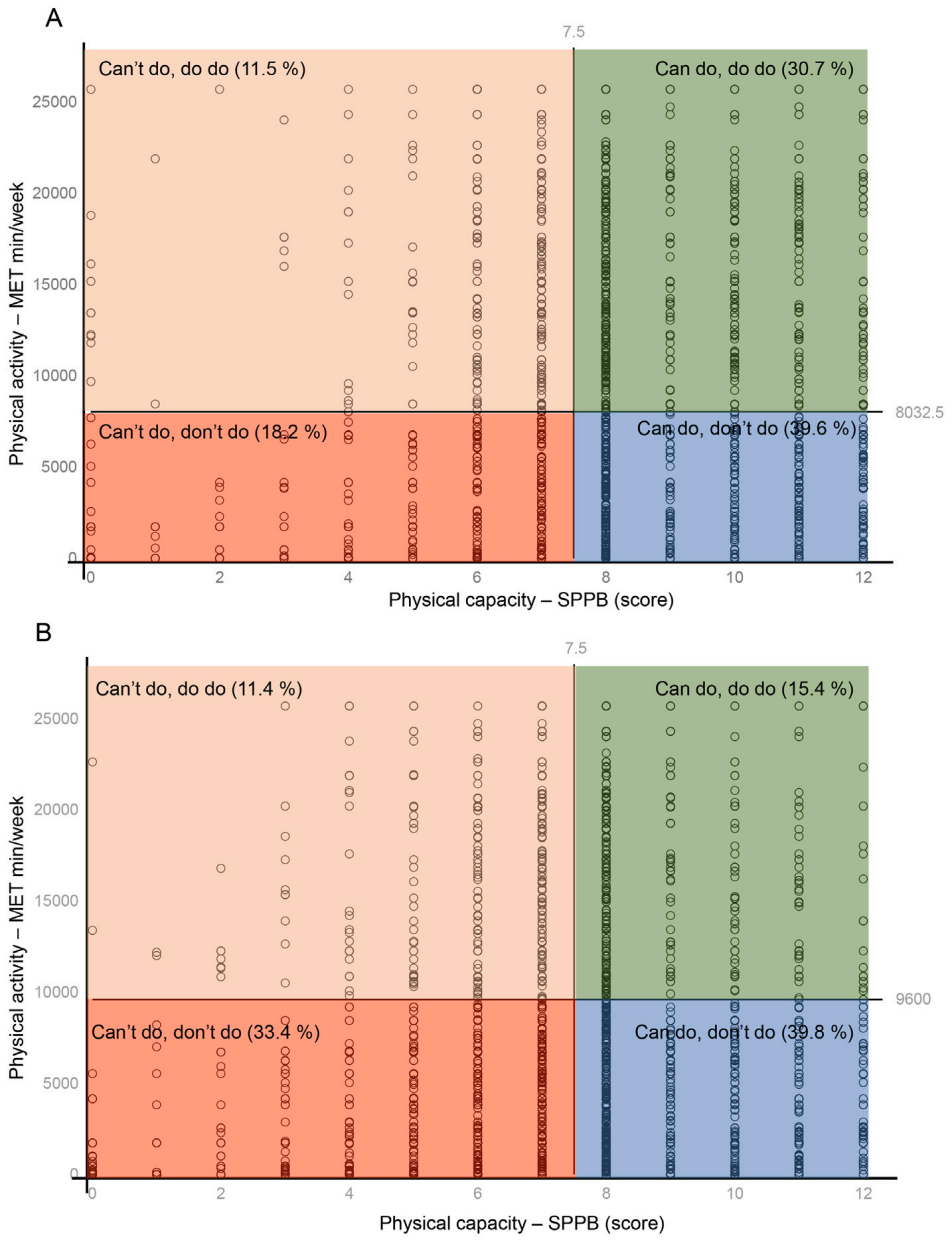
Graphical overview of the physical capacity-physical activity quadrant. **Panel A.** Men (n = 2577). **Panel B.** Women (n = 3034).

The PC-PA quadrant distribution differed between men and women. Specifically, 30.7% men were in the ‘can do, do do’ quadrant, 39.6% in the ‘can do, don’t do’ quadrant, 18.2% in the ‘can’t do, don’t do’ quadrant, and 11.5% in the ‘can’t do, do do’ quadrant, compared to 15.4%, 39.8%, 33.4%, and 11.4% of women, respectively (**Figure 1**, Panels A and B). We observed significant differences across quadrants for all covariates, except for sleep duration. Participants in the ‘can’t do, don’t do’ quadrant, which served as the reference, had a higher mean age, a higher proportion of women, lower percentages of individuals with a spouse, and lower scores in cognition and peak expiratory flow (**Table 1**).

**Table 1:** Baseline characteristics of the study population by PC-PA quadrants*

	Can do/do do (n = 1258)	Can do/don’t do (n = 2229)	Can’t do/don’t do (n = 1483)	Can’t do/do do (n = 641)	*P*-value†
**Age, x̄ (SD)**	58.0 (8.0)	60.2 (9.2)	61.2 (11.1)	55.5 (7.3)	<0.001
**Sex**					<0.001
Man	791 (62.9)	1022 (45.9)	469 (31.6)	295 (46.0)	
Woman	467 (37.1)	1207 (54.1)	1014 (68.4)	346 (54.0)	
**Marital status**					<0.001
With spouse	1167 (92.8)	1933 (86.7)	1199 (80.8)	588 (91.7)	
Without spouse	91 (7.2)	296 (13.3)	284 (19.2)	53 (8.3)	
**Residence**					<0.001
Urban	271 (21.5)	1046 (46.9)	603 (40.7)	164 (25.6)	
Rural	987 (78.5)	1183 (53.1)	880 (59.3)	477 (74.4)	
**Education**					<0.001
Elementary school or below	887 (70.5)	1439 (64.6)	1105 (74.5)	459 (71.6)	
Middle school	271 (21.5)	469 (21.0)	238 (16.0)	144 (22.5)	
High school or above	100 (7.9)	321 (14.4)	140 (9.4)	38 (5.9)	
**Medical insurance**					<0.001
No	57 (4.5)	203 (9.1)	145 (9.8)	39 (6.1)	
Yes	1201 (95.5)	2026 (90.9)	1338 (90.2)	602 (93.9)	
**Endowment insurance**					<0.001
No	1024 (81.4)	1533 (68.8)	1110 (74.8)	576 (89.9)	
Yes	234 (18.6)	696 (31.2)	373 (25.2)	65 (10.1)	
**Smoking history**					<0.001
No	631 (50.2)	1412 (63.3)	1062 (71.6)	383 (59.8)	
Yes	627 (49.8)	817 (36.7)	421 (28.4)	258 (40.2)	
**Drinking status**					<0.001
No	702 (55.8)	1527 (68.5)	1148 (77.4)	419 (65.4)	
Yes	556 (44.2)	702 (31.5)	335 (22.6)	222 (34.6)	
**Sleep duration**					0.053
≤6 h	615 (48.9)	1125 (50.5)	784 (52.9)	309 (48.2)	
6 ~ 8 h	536 (42.6)	955 (42.8)	586 (39.5)	271 (42.3)	
>8 h	107 (8.5)	149 (6.7)	113 (7.6)	61 (9.5)	
**Comorbidity**					<0.001
None	443 (35.2)	706 (31.7)	368 (24.8)	210 (32.8)	
1 condition	397 (31.6)	659 (29.6)	412 (27.8)	205 (32.0)	
≥2 conditions	418 (33.2)	864 (38.8)	703 (47.4)	226 (35.3)	
**BMI**					<0.001
<18.5 kg/m^2^	91 (7.2)	132 (5.9)	121 (8.2)	40 (6.2)	
18.5–24.0 kg/m^2^	786 (62.5)	1084 (48.6)	704 (47.5)	375 (58.5)	
24.0–28.0 kg/m^2^	299 (23.8)	741 (33.2)	440 (29.7)	172 (26.8)	
≥28.0 kg/m^2^	82 (6.5)	272 (12.2)	218 (14.7)	54 (8.4)	
**Cognition scores, x̄ (SD)**	15.7 (4.5)	16.0 (4.7)	14.5 (5.0)	15.5 (4.6)	<0.001
**Peak expiratory flow, x̄ (SD)**	322.5 (124.4)	294.5 (124.5)	246.0 (110.0)	288.7 (124.9)	<0.001

BMI – body mass index, SD – standard deviation, x̄ – mean

*Presented as n (%) unless specified otherwise.

†One-way ANOVA for continuous variables and χ^2^ tests for categorical variables.

### Associations of PC-PA quadrants with baseline depression

Compared to the reference group, both the ‘can do, do do’ (*β* = −0.930; 95% CI = −1.382, −0.477; *P* < 0.001) and ‘can do, don’t do’ (*β* = −1.630; 95% CI = −2.010, −1.249; *P* < 0.001) quadrants were associated with significantly lower baseline depression scores. We observed no significant association for the ‘can’t do, do do’ quadrant. These associations remained consistent in sex-stratified analyses, meaning there was no significant interaction effect by sex (**Table 2**).

**Table 2:** Associations of PC-PA quadrants with the baseline depression*

	*β* (95% CI)	SE	*P*-value
**All participants**			
Can do/do do	−0.930 (−1.382, −0.477)	0.231	<0.001
Can do/don’t do	−1.630 (−2.010, −1.249)	0.194	<0.001
Can’t do/do do	−0.286 (−0.254, 0.826)	0.275	0.299
Can’t do/don’t do	ref		
**Sex (*P*-value for interaction = 0.217)**			
**Male**			
Can do/do do	−0.769 (−1.408, −0.130)	0.326	0.018
Can do/don’t do	−1.346 (−1.940, −0.752)	0.303	<0.001
Can’t do/do do	−0.139 (−0.940, 0.662)	0.408	0.734
Can’t do/don’t do	ref		
**Female**			
Can do/do do	−1.081 (−1.751, −0.411)	0.342	0.002
Can do/don’t do	−1.830 (−2.331, −1.328)	0.256	<0.001
Can’t do/do do	−0.698 (−0.043, 1.440)	0.378	0.065
Can’t do/don’t do	ref		

CI – confidence interval, ref – reference, PA – physical activity, PC – physical capacity, SE – standard error, *β* – regression coefficient

*The *β* and *Р*-values are adjusted for age, sex, marital status, residence, education, medical insurance, endowment insurance, smoking history, drinking status, sleep duration, comorbidity, body mass index, cognition scores, and peak expiratory flow.

### Associations of PC-PA quadrants with depression progression

Among the 3589 participants in the longitudinal analysis of depression progression, 908 (25.3%) were in the ‘can do, do do’, 1432 (39.9%) in the ‘can do, don’t do’, 793 (22.1%) in the ‘can’t do, don’t do’, and 456 (12.7%) in the ‘can’t do, do do’ quadrant (Table S2 in the **Online Supplementary Document**). Compared with the ‘can’t do, don’t do’ reference group, participants in the two quadrants with preserved PC (‘can do, do do’ and ‘can do, don’t do’) had significantly lower levels of depressive symptoms at baseline, with *β* values of −0.857 (95% CI = −1.362, −0.352) and −1.474 (95% CI = −1.924, −1.025), respectively (**Table 3**). The non-significant overall time main effect (*P* = 0.634) suggests that depressive symptoms in the reference group did not exhibit a statistically significant linear change over the follow-up period.

**Table 3:** Associations of PC-PA quadrants with the depression progression*

	*β* (95% CI)	SE	*P*-value
**All participants**			
Can do/do do	−0.857 (−1.362, −0.352)	0.258	0.001
Can do/don’t do	−1.474 (−1.924, −1.025)	0.229	<0.001
Can’t do/do do	0.356 (−0.243, 0.954)	0.305	0.244
Can’t do/don’t do	ref		
Time, years	0.015 (−0.046, 0.076)	0.032	0.634
Can do/do do × time	0.102 (0.019, 0.186)	0.043	0.017
Can do/don’t do × time	0.133 (0.056, 0.209)	0.039	0.001
Can’t do/do do × time	0.061 (−0.040, 0.162)	0.052	0.238
Can’t do/don’t do × time	ref		
**Sex (*P*-value for interaction = 0.599)**			
**Male**			
Can do/do do	−0.751 (−1.484, −0.019)	0.374	0.044
Can do/don’t do	−1.186 (−1.902, −0.470)	0.365	0.001
Can’t do/do do	0.298 (−0.596, 1.193)	0.456	0.514
Can’t do/don’t do	ref		
Time, years	−0.001 (−0.105, 0.103)	0.053	0.985
Can do/do do × time	0.093 (−0.030, 0.217)	0.063	0.138
Can do/don’t do × time	0.108 (−0.013, 0.229)	0.062	0.081
Can’t do/do do × time	0.094 (−0.058, 0.246)	0.077	0.225
Can’t do/don’t do × time	ref		
**Female**			
Can do/do do	−0.957 (−1.691, −0.223)	0.375	0.011
Can do/don’t do	−1.705 (−2.293, −1.116)	0.300	<0.001
Can’t do/do do	0.480 (−0.335, 1.295)	0.416	0.248
Can’t do/don’t do	ref		
Time, years	0.021 (−0.056, 0.098)	0.039	0.591
Can do/do do × time	0.134 (0.010, 0.258)	0.063	0.035
Can do/don’t do × time	0.158 (0.058, 0.259)	0.051	0.002
Can’t do/do do × time	0.041 (−0.098, 0.179)	0.071	0.562
Can’t do/don’t do × time	ref		

CI – confidence interval, PA – physical activity, PC – physical capacity, ref – reference, SE – standard error, *β* – regression coefficient

*The *β* and *Р*-values are adjusted for age, sex, marital status, residence, education, medical insurance, endowment insurance, smoking history, drinking status, sleep duration, comorbidity, body mass index, cognition scores, and peak expiratory flow.

**Figure 2:**
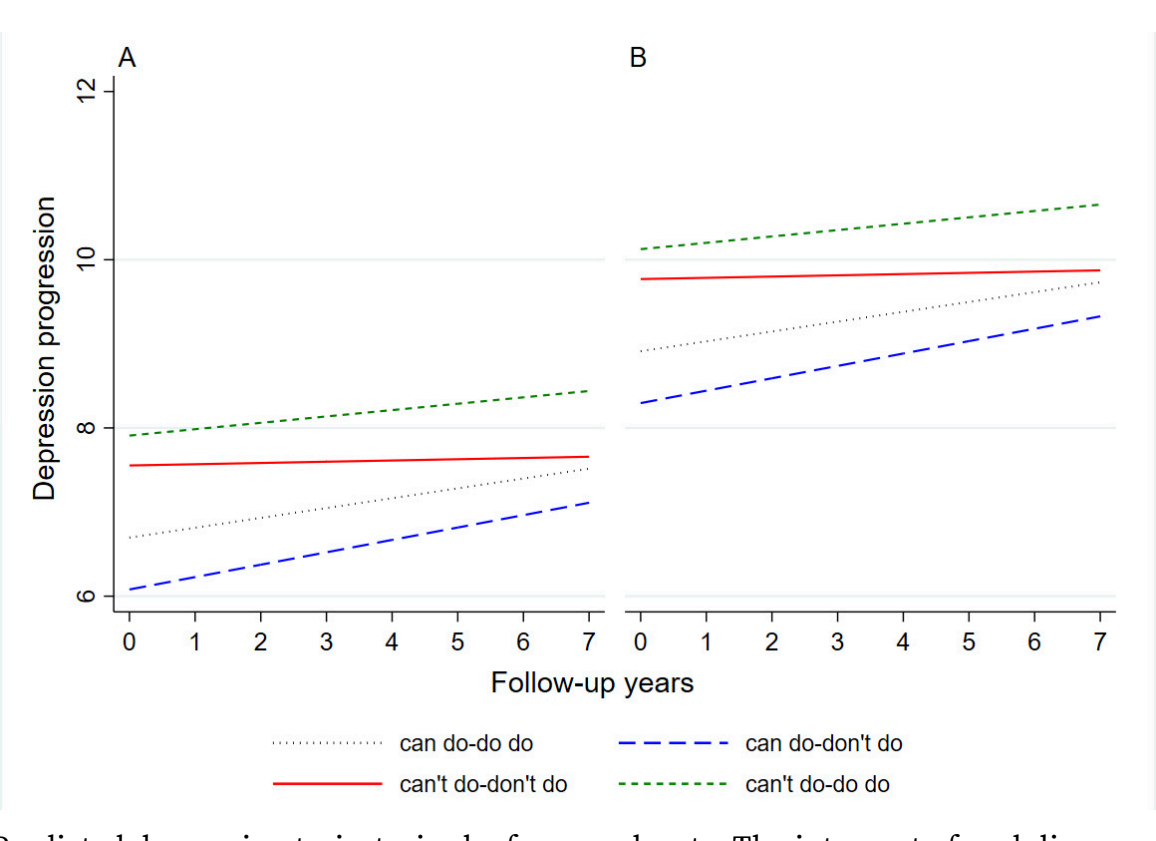
Predicted depression trajectories by four quadrants. The intercept of each line represents the baseline depression. The slope of each line represents the annual depression increase. **Panel A.** Men (n = 2577). **Panel B.** Women (n = 3034).

While these baseline differences were consistent in both men and women subgroups in stratified analyses, we noted significant sex differences in the associations between PC-PA quadrants and changes in depressive symptoms over time (**Figure 2**). Specifically, we observed no significant differences in the rate of change in depressive symptoms across quadrants over time, suggesting broadly similar and stable trajectories of depressive symptoms across quadrants. In contrast, women in both the ‘can do, do do’ (*β* = 0.134; 95% CI = 0.010, 0.258) and ‘can do, don’t do’ (*β* = 0.158; 95% CI = 0.058, 0.259) quadrants showed significantly steeper increases in depressive symptoms over time compared to the reference group, despite their initially lower symptom burden.

Supplementary analyses across age and residential settings showed consistent associations with the main results, as did the sensitivity analyses using alternative PA and PC thresholds, and those excluding participants with cardiovascular or pulmonary diseases (Tables S3–6 in the **Online Supplementary Document**).

## DISCUSSION

To our knowledge, this is the first study to examine how PC and PA, jointly classified using a quadrant-based approach, relate to longitudinal trajectories of depressive symptoms in middle-aged and older adults. Using thresholds derived from a CHARLS cohort, we found preserved PC to be associated with lower depressive symptom levels at baseline, including among individuals with low PA. Notably, longitudinal patterns of depressive symptoms differed by sex, with distinct patterns observed in women.

Previous studies have established the protective effects of PC and PA in reducing depressive symptoms [6,7], but have mainly examined PA and PC independently, overlooking their combined effects and potential interactions. To address this, we used a quadrant-based approach to categorise PC and PA combinations and gain insights on their impact on depression. We found that PC and PA do not always agree, with some patients maintaining enough PA despite limited PC, and others with adequate PC exhibiting low PA. This indicates that different combinations of PC and PA are associated with distinct baseline levels in general and different longitudinal patterns of depressive symptoms in women specifically.

Our findings indicate that preserved PC is associated with lower depressive symptom levels. In the overall sample, participants in the ‘can do, don’t do’ quadrant had CESD-10 scores that were approximately had 1.47 points lower scores at baseline than the reference group, while those in the ‘can do, do do’ quadrant showed a difference of approximately 0.86 points. Although these effect sizes are clinically modest in relation to the full CESD-10 scale range (0–30), they may reflect meaningful differences in average depressive symptom burden at the population level. Notably, participants in the ‘can’t do, do do’ quadrant did not show significantly lower depressive symptom levels compared with the reference group, suggesting that PA alone may be insufficiently associated with lower depressive symptoms when PC is compromised. Previous evidence suggests that the mental health benefits of PA may depend on an individual’s capacity to engage in and sustain these activities over time [27,28]. Functional independence and physiological reserve associated with preserved PC have been linked to better mental well-being, including lower depressive symptoms, in prior research [29].

Although our formal test for sex interaction was not statistically significant, sex-stratified models suggested different longitudinal patterns. Slopes did not differ across quadrants in men, whereas women in preserved PC quadrants exhibited steeper increases over time relative to the reference group. The change aligns with literature indicating that women may be more sensitive to psychological stressors and hormonal changes, which may influence the course of depressive symptoms over time [30]. Factors such as societal expectations and caregiving roles could contribute to this sex-specific trend [31].

The association between PC, PA, and depressive symptom trajectories likely reflects an interaction between biological, psychosocial, and socioeconomic mechanisms. From a biological perspective, both PA and PC are known to regulate key neurotransmitters, such as serotonin, dopamine, and endorphins, which are essential for mood stabilisation [32]. Individuals with higher PC can maintain regular PA more effectively, thereby promoting neurotransmitter release and reducing depressive symptoms [33]. Regular exercise may also mitigate systemic inflammation by lowering levels of markers like C-reactive protein, which has been associated with depression [34]. From a psychosocial perspective, preserved PC contributes to greater functional independence, potentially enhancing mental health and well-being. Higher PC levels may foster self-efficacy, particularly in older adults, empowering them with proactive coping strategies and a sense of control and resilience against stress [35]. Additionally, the ability to engage in social activities facilitated by good physical function may also expand social support networks, providing crucial buffers against depression [36,37]. Socioeconomic contexts may further shape this pathway, as individuals with higher socioeconomic status may have greater access to PA resources, safer environments, and infrastructure, thereby facilitating the translation of PC into actual activity engagement [38].

Our findings highlight the importance of assessing both PC and PA when monitoring and preventing depressive symptoms. Routine evaluation of PC could help identify individuals at higher risk of depression, allowing for early and targeted interventions. Personalised strategies focusing on maintaining or improving PC, such as strength training and balance exercises, might be effectively incorporated into a comprehensive depression management plan [39]. Future research, including randomised controlled trials, should investigate whether health education and counselling could effectively promote PA in the ‘can do, don’t do’ group, and whether functional training could benefit the ‘can’t do, don’t do’ group [12]. The observed sex differences indicate that strategies may need to be tailored for women, who may be more vulnerable to changes in depressive symptoms over time [40]. Incorporating these interventions into community health programmes could facilitate early intervention and potentially reduce the burden of depression, and a multidisciplinary approach involving mental health professionals and physical trainers may further enhance the effectiveness of these interventions.

Our findings have several important implications for practice. First, routine assessment of PC could be integrated into regular nursing assessments for older adults, enabling identification of individuals at heightened risk due to declining functional ability. Second, community and primary care nurses are well-positioned to provide education, motivation, and support to enhance both physical capacity and activity levels. Nurse-led interventions, such as strength and balance training programmes, home-based exercise coaching, and behavioural counselling, could help individuals in the ‘can do, don’t do’ quadrant increase their activity levels, while targeted functional training may assist those in the ‘can’t do, don’t do’ group in regaining independence. Finally, incorporating capacity-based screening and tailored interventions into community nursing practice may help reduce the psychological burden of ageing and promote active, healthy ageing.

Our study has several strengths. The novel PC-PA quadrant classification enabled us to examine the combined effects of PC and PA on depression progression, providing actionable insights for targeted interventions, while the longitudinal analysis based on the CHARLS data set allowed us to dynamically track changes in depressive symptoms over time. However, several limitations should be noted. First, both PA and depressive symptoms were self-reported, which, despite their collection using validated instruments, may introduce recall bias and subjective misestimation, potentially influencing the results. Second, assessments of PC and PA were limited to baseline, with no subsequent measurements during follow-up. The failure to capture potential changes over time may have led to exposure misclassification. Third, our longitudinal analyses were restricted to participants with complete depressive symptom data across all follow-up waves, which may have introduced selection bias if missingness was related to depression severity or health status. In addition, residual confounding cannot be excluded. Although we adjusted for a range of factors, unmeasured or imperfectly measured variables, such as medication use, dietary patterns, psychosocial stress, or more detailed socioeconomic circumstances, may have influenced the observed associations. Finally, given that the study population consisted of middle-aged and older adults in China, the generalisability of our results to other age groups, populations, or cultural contexts is limited.

## CONCLUSIONS

In our study, preserved PC was associated with lower depressive symptom levels in middle-aged and older adults, including those with low PA. However, longitudinal trajectories differed by sex, with women showing a steeper increase in depressive symptoms over time despite lower baseline levels. These findings highlight the importance of incorporating PC assessment and sex-specific considerations into mental health monitoring in ageing populations.

## Additional material


Online Supplementary Document
