# Supplementary material for: Joint effect of physical capacity and physical activity predicts depression progression in middle-aged and older Chinese adults
Source: J Glob Health. 2026 Mar 27;16:04093. doi: 10.7189/jogh.16.04093 (PMC13021057; doi:10.7189/jogh.16.04093)
Supplement: Online Supplementary Document [file jogh-16-04093-s001.pdf]

Supplement to: Ding X, Zhou H, Zhang R, Wu W, Chen Y. Joint effect of physical capacity and physical activity predicts depression progression in middle-aged and older Chinese adults. J Glob Health. 2026;16:04093.

Checklist S1 STROBE Statement—Checklist of items that should be included in reports of cohort studies

Figure S1 Flowchart of the enrollment of this study population

Table S1 ROC curve parameters for the prediction of depression for men (n = 2594) and women (n = 3046)

Table S2 Characteristics of the study population of the depression progression analysis by PC-PA quadrants

Table S3 Stratification analysis of associations between PC-PA quadrants and the baseline depression

Table S4 Stratification analysis of associations between PC-PA quadrants and the depression progression

Table S5 Sensitivity analysis using WHO PA thresholds and SPPB score of 9 for PC

Table S6 Sensitivity analysis excluding participants with cardiovascular or pulmonary diseases

Text S1 Explanation of authorship change statement

Checklist S1 STROBE Statement—Checklist of items that should be included in reports of ***cohort studies***

	Item No	Recommendation	Page No
**Title and abstract**	1	(*a*) Indicate the study's design with a commonly used term in the title or the abstract	1
		(*b*) Provide in the abstract an informative and balanced summary of what was done and what was found	1-2
**Introduction**			
Background/rationale	2	Explain the scientific background and rationale for the investigation being reported	2-3
Objectives	3	State specific objectives, including any prespecified hypotheses	3
**Methods**			
Study design	4	Present key elements of study design early in the paper	3
Setting	5	Describe the setting, locations, and relevant dates, including periods of recruitment, exposure, follow-up, and data collection	4
Participants	6	(*a*) Give the eligibility criteria, and the sources and methods of selection of participants. Describe methods of follow-up	3-4
		(*b*) For matched studies, give matching criteria and number of exposed and unexposed	NA
Variables	7	Clearly define all outcomes, exposures, predictors, potential confounders, and effect modifiers. Give diagnostic criteria, if applicable	4-5
Data sources/ measurement	8*	For each variable of interest, give sources of data and details of methods of assessment (measurement). Describe comparability of assessment methods if there is more than one group	4-5
Bias	9	Describe any efforts to address potential sources of bias	7-8
Study size	10	Explain how the study size was arrived at	NA
Quantitative variables	11	Explain how quantitative variables were handled in the analyses. If applicable, describe which groupings were chosen and why	7
Statistical methods	12	(*a*) Describe all statistical methods, including those used to control for confounding	7
		(*b*) Describe any methods used to examine subgroups and interactions	8
		(*c*) Explain how missing data were addressed	3
		(*d*) If applicable, explain how loss to follow-up was addressed	3
		(*e*) Describe any sensitivity analyses	8
**Results**			
Participants	13*	(a) Report numbers of individuals at each stage of study—eg numbers potentially eligible, examined for eligibility, confirmed eligible, included in the study, completing follow-up, and analysed	8
		(b) Give reasons for non-participation at each stage	8
		(c) Consider use of a flow diagram	8
Descriptive data	14*	(a) Give characteristics of study participants (eg demographic, clinical, social) and information on exposures and potential confounders	8
		(b) Indicate number of participants with missing data for each variable of interest	8
		(c) Summarise follow-up time (eg, average and total amount)	NA
Outcome data	15*	Report numbers of outcome events or summary measures over time	9

Main results	16	(*a*) Give unadjusted estimates and, if applicable, confounder-adjusted estimates and their precision (eg, 95% confidence interval). Make clear which confounders were adjusted for and why they were included	9
		(*b*) Report category boundaries when continuous variables were categorized	6
		(*c*) If relevant, consider translating estimates of relative risk into absolute risk for a meaningful time period	NA
Other analyses	17	Report other analyses done—eg analyses of subgroups and interactions, and sensitivity analyses	9
**Discussion**			
Key results	18	Summarise key results with reference to study objectives	10
Limitations	19	Discuss limitations of the study, taking into account sources of potential bias or imprecision. Discuss both direction and magnitude of any potential bias	13
Interpretation	20	Give a cautious overall interpretation of results considering objectives, limitations, multiplicity of analyses, results from similar studies, and other relevant evidence	10-12
Generalisability	21	Discuss the generalisability (external validity) of the study results	13
**Other information**			
Funding	22	Give the source of funding and the role of the funders for the present study and, if applicable, for the original study on which the present article is based	14

*Give information separately for exposed and unexposed groups.

Note: An Explanation and Elaboration article discusses each checklist item and gives methodological background and published examples of transparent reporting. The STROBE checklist is best used in conjunction with this article (freely available on the Web sites of PLoS Medicine at http://www.plosmedicine.org/, Annals of Internal Medicine at http://www.annals.org/, and Epidemiology at http://www.epidem.com/). Information on the STROBE Initiative is available at http://www.strobe-statement.org.

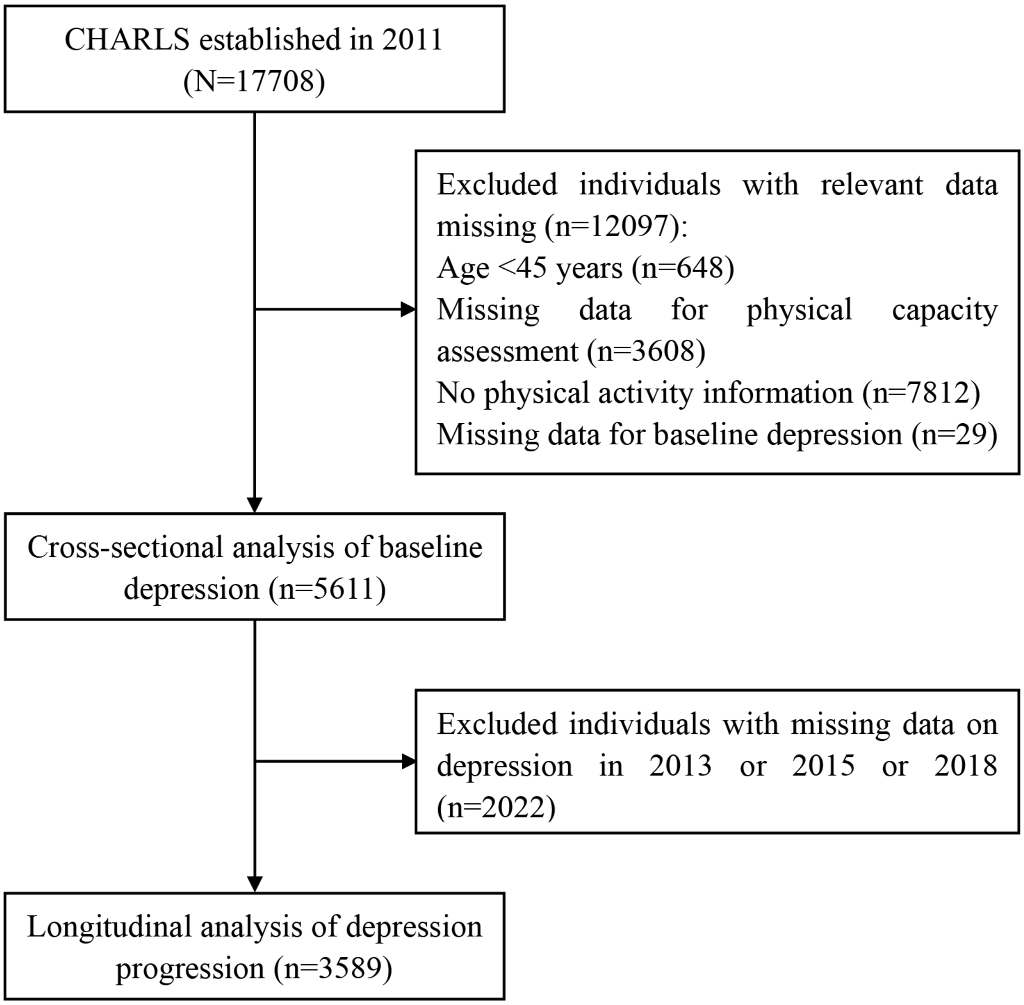

Figure S1 Flowchart of the enrollment of this study population

Table S1 ROC curve parameters for the prediction of depression for men (n = 2577) and women (n = 3034).

Dependent Variable	Area Under ROC Curve	Sensitivity	Specificity
Physical activity, MET-minutes/week			
Men	0.748 (0.723-0.769)	0.718	0.677
Women	0.714 (0.695-0.732)	0.594	0.725
Physical capacity, SPPB score			
Men	0.750 (0.727-0.773)	0.755	0.647
Women	0.719 (0.701-0.738)	0.757	0.577

ROC = receiver operating characteristic; SPPB = short physical performance battery.

Table S2 Characteristics of the study population of the depression progression analysis by PC-PA quadrants

Factor	Can do-do do	Can do-don't do	Can't do-don't do	Can't do-do do	*P* value
N, n (%)	908 (25.3)	1432 (39.9)	793 (22.1)	456 (12.7)	
Age, mean (SD)	57.5 (7.7)	59.2 (8.6)	57.8 (8.4)	55.2 (6.7)	<0.001
Sex, n (%)					<0.001
Man	551 (60.7)	629 (43.9)	227 (28.6)	201 (44.1)	
Woman	357 (39.3)	803 (56.1)	566 (71.4)	255 (55.9)	
Marital status, n (%)					<0.001
With spouse	841 (92.6)	1277 (89.2)	690 (87.0)	424 (93.0)	
Without spouse	67 (7.4)	155 (10.8)	103 (13.0)	32 (7.0)	
Residence, n (%)					<0.001
Urban	192 (21.1)	610 (42.6)	306 (38.6)	101 (22.1)	
Rural	716 (78.9)	822 (57.4)	487 (61.4)	355 (77.9)	
Education, n (%)					<0.001
Elementary school or below	649 (71.5)	930 (64.9)	559 (70.5)	322 (70.6)	
Middle school	185 (20.4)	325 (22.7)	148 (18.7)	105 (23.0)	
High school or above	74 (8.1)	177 (12.4)	86 (10.8)	29 (6.4)	
Medical insurance, n (%)					0.001
No	39 (4.3)	118 (8.2)	51 (6.4)	23 (5.0)	
Yes	869 (95.7)	1314 (91.8)	742 (93.6)	433 (95.0)	
Endowment insurance, n (%)					<0.001
No	743 (81.8)	1028 (71.8)	633 (79.8)	408 (89.5)	
Yes	165 (18.2)	404 (28.2)	160 (20.2)	48 (10.5)	
Smoking history, n (%)					<0.001
No	479 (52.8)	948 (66.2)	586 (73.9)	280 (61.4)	
Yes	429 (47.2)	484 (33.8)	207 (26.1)	176 (38.6)	
Drinking status, n (%)					<0.001
No	507 (55.8)	1008 (70.4)	618 (77.9)	296 (64.9)	

Yes	401 (44.2)	424 (29.6)	175 (22.1)	160 (35.1)	
Sleep duration, n (%)					0.45
≤6 hours	438 (48.2)	709 (49.5)	404 (50.9)	221 (48.5)	
6~8 hours	394 (43.4)	629 (43.9)	331 (41.7)	193 (42.3)	
>8 hours	76 (8.4)	94 (6.6)	58 (7.3)	42 (9.2)	
Comorbidity, n (%)					<0.001
None	323 (35.6)	460 (32.1)	207 (26.1)	152 (33.3)	
1 condition	288 (31.7)	411 (28.7)	228 (28.8)	148 (32.5)	
≥2 conditions	297 (32.7)	561 (39.2)	358 (45.1)	156 (34.2)	
Body mass index, n (%)					<0.001
<18.5 kg/m^2	59 (6.5)	75 (5.2)	43 (5.4)	24 (5.3)	
18.5-24.0 kg/m^2	567 (62.4)	686 (47.9)	373 (47.0)	263 (57.7)	
24.0-28.0 kg/m^2	220 (24.2)	484 (33.8)	248 (31.3)	129 (28.3)	
≥28.0 kg/m^2	62 (6.8)	187 (13.1)	129 (16.3)	40 (8.8)	
Cognition scores, mean (SD)	15.7 (4.6)	16.0 (4.6)	15.3 (4.7)	15.6 (4.7)	0.004
Peak expiratory flow, mean (SD)	322.1 (125.3)	298.5 (122.1)	264.4 (109.1)	287.2 (123.7)	<0.001

Comparisons of the characteristics across four quadrants are performed with one-way ANOVA for continuous variables and χ^2 tests for categorical variables.

Table S3 Stratification analysis of associations between PC-PA quadrants and the baseline depression

Quadrant	*β* (95% CI)	Std. Err.	*P* value
Age (*P* for interaction=0.302)			
Age <60 years			
Can do-do do	-0.851 (-1.442 to -0.260)	0.301	0.005
Can do-don't do	-1.370 (-1.891 to -0.849)	0.266	<0.001
Can't do-do do	0.496 (-1.130 to 1.122)	0.319	0.120
Can't do-don't do	Reference	—	—
Age ≥60 years			
Can do-do do	-1.105 (-1.809 to -0.401)	0.359	0.002
Can do-don't do	-2.012 (-2.586 to -1.439)	0.292	<0.001
Can't do-do do	0.196 (-0.900 to 1.293)	0.559	0.726
Can't do-don't do	Reference	—	—
Residence (*P* for interaction=0.102)			
Urban			
Can do-do do	-0.448 (-1.253 to 0.376)	0.411	0.276
Can do-don't do	-1.536 (-2.090 to -0.983)	0.282	<0.001
Can't do-do do	0.637 (-0.308 to 1.582)	0.482	0.186
Can't do-don't do	Reference	—	—
Rural			
Can do-do do	-1.094 (-1.654 to -0.535)	0.285	<0.001
Can do-don't do	-1.687 (-2.203 to -1.170)	0.263	<0.001
Can't do-do do	0.065 (-0.603 to 0.733)	0.341	0.849
Can't do-don't do	Reference	—	—

The *β* and *P* values are adjusted for age, sex, marital status, residence, education, medical insurance, endowment insurance, smoking history, drinking status, sleep duration, comorbidity, body mass index, cognition scores, and peak expiratory flow.

Table S4 Stratification analysis of associations between PC-PA quadrants and the depression progression

Quadrant	*β* (95% CI)	Std. Err.	*P* value
Age (*P* for interaction=0.715)			
Age <60 years			
Time, years	0.090 (0.017 to 0.163)	0.037	0.016
"Can do-do do" × time	0.077 (-0.028 to 0.182)	0.054	0.151
"Can do-don't do" × time	0.066 (-0.031 0.164)	0.050	0.184
"Can't do-do do" × time	0.004 (-0.111 to 0.120)	0.059	0.938
"Can't do-don't do" × time	Reference	—	—
Age ≥60 years			
Time, years	-1.152 (-0.262 to -0.041)	0.056	0.007
"Can do-do do" × time	0.203 (0.062 to 0.344)	0.072	0.005
"Can do-don't do" × time	0.291 (0.163 to 0.419)	0.065	0.001
"Can't do-do do" × time	0.147 (-0.071 to 0.364)	0.111	0.187
"Can't do-don't do" × time	Reference	—	—
Residence (*P* for interaction=0.102)			
Urban			
Time, years	-0.034 (-0.126 to 0.058)	0.047	0.464
"Can do-do do" × time	0.069 (-0.079 to 0.217)	0.075	0.359
"Can do-don't do" × time	0.112 (0.000 to 0.225)	0.057	0.050
"Can't do-do do" × time	0.064 (-0.120 to 0.248)	0.094	0.496
"Can't do-don't do" × time	Reference	—	—
Rural			
Time, years	0.046 (-0.035 to 0.126)	0.410	0.265
"Can do-do do" × time	0.093 (-0.011 to 0.198)	0.053	0.079
"Can do-don't do" × time	0.153 (0.051 to 0.254)	0.052	0.003
"Can't do-do do" × time	0.043 (-0.081 to 0.167)	0.063	0.495
"Can't do-don't do" × time	Reference	—	—

The *β* and *P* values are adjusted for age, sex, marital status, residence, education, medical insurance, endowment insurance, smoking history, drinking status, sleep duration, comorbidity, body mass index, cognition scores, and peak expiratory flow.

Table S5 Sensitivity analysis using WHO PA thresholds and SPPB score of 9 for PC

Quadrant	*β* (95% CI)	Std. Err.	*P* value
All participants			
Can do-do do	-0.774 (-1.343 to -0.205)	0.290	0.008
Can do-don't do	-1.446 (-2.126 to -0.767)	0.347	<0.001
Can't do-do do	-0.206 (-0.636 to 0.223)	0.219	0.347
Can't do-don't do	Reference	—	—
Time, years	0.049 (-0.010 to 0.108)	0.030	0.101
"Can do-do do" × time	0.065 (-0.026 to 0.156)	0.046	0.164
"Can do-don't do" × time	0.090 (-0.021 to 0.200)	0.056	0.113
"Can't do-do do" × time	0.065 (-0.006 to 0.137)	0.037	0.074
"Can't do-don't do" × time	Reference	—	—
Sex (*P* for interaction=0.017)			
Man			
Can do-do do	-0.624 (-1.401 to 0.152)	0.396	0.115
Can do-don't do	-0.726 (-1.668 to 0.216)	0.481	0.131
Can't do-do do	-0.209 (-0.849 to 0.431)	0.327	0.522
Can't do-don't do	Reference	—	—
Time, years	0.041 (-0.050 to 0.132)	0.047	0.380
"Can do-do do" × time	0.083 (-0.040 to 0.205)	0.063	0.186
"Can do-don't do" × time	0.021 (-0.130 to 0.172)	0.077	0.788
"Can't do-do do" × time	0.048 (-0.059 to 0.155)	0.055	0.381
"Can't do-don't do" × time	Reference	—	—
Woman			
Can do-do do	-0.991 (-1.838 to -0.144)	0.432	0.022
Can do-don't do	-2.19 (-3.167 to -1.220)	0.497	<0.001
Can't do-do do	-0.173 (-0.754 to 0.407)	0.296	0.559
Can't do-don't do	Reference	—	—
Time, years	0.054 (-0.024 to 0.132)	0.040	0.175
"Can do-do do" × time	0.046 (-0.094 to 0.186)	0.071	0.518
"Can do-don't do" × time	0.162 (0.001 to 0.323)	0.082	0.049
"Can't do-do do" × time	0.081 (-0.016 to 0.178)	0.050	0.102
"Can't do-don't do" × time	Reference	—	—

The *β* and *P* values are adjusted for age, sex, marital status, residence, education, medical insurance, endowment insurance, smoking history, drinking status, sleep duration, comorbidity, body mass index, cognition scores, and peak expiratory flow.

Table S6 Sensitivity analysis excluding participants with cardiovascular or pulmonary diseases

Quadrant	*β* (95% CI)	Std. Err.	*P* value
All participants			
Can do-do do	-0.636 (-1.203 to -0.068)	0.290	0.028
Can do-don't do	-1.370 (-1.886 to -0.853)	0.264	<0.001
Can't do-do do	0.428 (-0.243 to 1.100)	0.342	0.211
Can't do-don't do	Reference	—	—
Time, years	0.059 (-0.011 to 0.128)	0.035	0.097
"Can do-do do" × time	0.062 (-0.030 to 0.155)	0.047	0.186
"Can do-don't do" × time	0.106 (0.020 to 0.192)	0.044	0.016
"Can't do-do do" × time	0.003(-0.108 to 0.114)	0.057	0.960
"Can't do-don't do" × time	Reference	—	—
Sex (*P* for interaction=0.237)			
Man			
Can do-do do	-0.413 (-1.240 to 0.413)	0.422	0.327
Can do-don't do	-0.878 (-1.700 to -0.056)	0.419	0.036
Can't do-do do	0.590 (-0.407 to 1.588)	0.509	0.246
Can't do-don't do	Reference	—	—
Time, years	0.0260 (-0.094 to 0.146)	0.061	0.672
"Can do-do do" × time	0.060 (-0.079 to 0.200)	0.071	0.398
"Can do-don't do" × time	0.078 (-0.061 to 0.218)	0.07	0.274
"Can't do-do do" × time	0.046 (-0.123 to 0.215)	0.086	0.592
"Can't do-don't do" × time	Reference	—	—
Woman			
Can do-do do	-0.814 (-1.639 to 0.011)	0.421	0.053
Can do-don't do	-1.723 (-2.402 to -1.043)	0.346	<0.001
Can't do-do do	0.417 (-0.503 to 1.337)	0.469	0.374
Can't do-don't do	Reference	—	—
Time, years	0.071 (-0.016 to 0.158)	0.044	0.108
"Can do-do do" × time	0.104 (-0.032 to 0.239)	0.069	0.133
"Can do-don't do" × time	0.140 (0.027 to 0.253)	0.058	0.015
"Can't do-do do" × time	-0.018 (-0.170 to 0.133)	0.077	0.813
"Can't do-don't do" × time	Reference	—	—

The *β* and *P* values are adjusted for age, sex, marital status, residence, education, medical insurance, endowment insurance, smoking history, drinking status, sleep duration, comorbidity, body mass index, cognition scores, and peak expiratory flow.

Text S1. Explanation of authorship change statement

The authors have requested changes to the order in the authorship byline after submission. Specifically, the initial order was as follows: Hui Zhou, Roulin Zhang, Xiong Ding, Weiqiang Wu, Yanjuan Chen. Here, Yanjuan Chen was assigned as the corresponding author.

After the revision process, the authors jointly agreed to revise the authorship byline as follows: Xiong Ding, Hui Zhou, Roulin Zhang, Weiqiang Wu, Yanjuan Chen. Here, Xioning Ding and Hui Zhou shared joint first authorship, while Weiqiang Wu and Yanjuan Chen were both acknowledged as corresponding authors.

The authors note that this change emerged due to the authors' contributions during the data analysis and manuscript development stages following peer review. They have provided an signed authorship form, a prior version of the manuscript, analytical code, and proof of correspondence between co-authors as evidence behind this change.
